# Acute Toxicities During Proton Therapy with or Without Simultaneous Chemotherapy in Pediatric CNS Tumors: A Retrospective Cohort Study

**DOI:** 10.3390/cancers18050859

**Published:** 2026-03-07

**Authors:** Eicke Schuermann, Sarah Peters, Jonas E. Adolph, Julien Merta, Stefan Rutkowski, Michael C. Frühwald, Philipp Dammann, Hermann L. Müller, Christof M. Kramm, Gudrun Fleischhack, Beate Timmermann, Stephan Tippelt

**Affiliations:** 1 Department of Pediatrics III, Center for Translational Neuro- and Behavioral Sciences (CTNBS), University Hospital of Essen, 45122 Essen, Germany; jonas.adolph@uk-essen.de (J.E.A.); gudrun.fleischhack@uk-essen.de (G.F.); 2 Department of Pediatric and Adolescent Medicine, Faculty of Medicine, University Hospital Cologne, University of Cologne, Kerpener Str. 62, 50937 Cologne, Germany; 3 West German Proton Therapy Center Essen (WPE), University Hospital Essen, 45122 Essen, Germany; sarah.peters@uk-essen.de (S.P.); julien.merta@uk-essen.de (J.M.); beate.timmermann@uk-essen.de (B.T.); 4 Clinic for Particle Therapy, University Hospital Essen, 45122 Essen, Germany; 5 West German Cancer Center (WTZ), 45122 Essen, Germany; 6 German Cancer Consortium (DKTK), 69120 Heidelberg, Germany; 7 Department of Pediatric Hematology and Oncology, Center for Obstetrics and Pediatrics, University Medical Center Hamburg-Eppendorf, 20251 Hamburg, Germany; s.rutkowski@uke.de; 8 Swabian Children’s Cancer Center, Pediatric and Adolescent Medicine, Faculty of Medicine, University Augsburg, 86156 Augsburg, Germany; michael.fruehwald@uk-augsburg.de; 9 Department of Neurosurgery and Spine Surgery, University Hospital Essen, 45122 Essen, Germany; philipp.dammann@uk-essen.de; 10 Department of Pediatrics and Pediatric Hematology/Oncology, University Children’s Hospital, Klinikum Oldenburg AöR, 26133 Oldenburg, Germany; mueller.hermann@klinikum-oldenburg.de; 11 Department of Child and Adolescent Health, Division of Pediatric Hematology and Oncology, University Medical Center Göttingen, 37075 Göttingen, Germany; christof.kramm@med.uni-goettingen.de

**Keywords:** simultaneous radiochemotherapy, proton beam therapy, safety, high grade adverse events, children, adolescents, CNS tumors

## Abstract

**Background:** Proton beam therapy (PBT) is a valuable alternative to photon radiotherapy of CNS tumors in children and adolescents. While most recent studies deal with the outcome or long-term side effects of PBT, the aim of this study was to investigate the feasibility of PBT with a particular focus on the acute toxicity of a simultaneous radiochemotherapy (sPBCT). **Patients and methods:** We enrolled 199 children [median age 7.4 years (range, 0.9–17.9)], who received altogether 200 courses of PBT/sPBCT at initial diagnosis (*n* = 121) or at relapse (*n* = 79) with sPBCT in 52 (26%) courses. Data collection to PBT/sPBCT was based on the medical records and the KiProReg (Registry study of Standard Proton Therapy in Children at West German Proton Therapy Center) with a primarily descriptive-statistical and logistic regression analysis. **Results:** During PBT/sPBCT a total of *n* = 704 adverse events (AEs, mean 3.4 per course) were observed. Eighty-seven of them were graded as high-grade adverse events (HGAEs, Common Terminology Criteria for Adverse Eventº ≥3 (CTCAE)) which occurred in 67 (33.5%) PBT/sPBCT courses. HGAEs were in particular hematotoxicity (*n* = 43; 64.1%) and infections (*n* = 18; 26.8%). A significantly higher rate of HGAEs was documented in patients treated with sPBCT (*n* = 33/52; 63.5%) compared to those with PBT only (*n* = 34/148; 23.0%) (*p* = 0.001). In children with sPBCT, 15 (28.8%) patients could not receive the recommended dose or schedule of the planned chemotherapy (CTx) due to HGAEs, with the rate of planned CTx courses performed being significantly lower in patients receiving intensive intravenous CTx (*p* < 0.001). Interruptions of PBT and of simultaneous CTx were both significantly associated with the occurrence of infections [Odds ratios 3.002 (95% CI 1.005–8.971, *p* = 0.049) and 3.905 (95% CI 1.005–15.174, *p* = 0.049)]. Total discontinuation of treatment did not occur. **Conclusions:** Concurrent CTx during proton therapy is associated with a significant increased risk for HGAE occurrence and therapy interruptions requiring individual dose and schedule adjustments dependent on CTx intensity, very experienced interdisciplinary teams as well as intensive care and in-/out-patient oncology facilities on site.

## Simple Summary

Simultaneous chemotherapy (sCTx) with radiotherapy has been used for several decades in the treatment of primary CNS tumors in children and adolescents mostly in curative intention and with the aim to improve local tumor control by radiosensitizing effects and in order to retain systemic tumor control in- and outside the CNS. The technique of proton therapy, as an alternative to classical photon therapy, allows high-precision radiotherapy as part of a multimodal treatment in primary CNS tumors in childhood. The purpose of our study was to evaluate the acute toxicity and to demonstrate the feasibility during proton therapy with or without sCTx in children and adolescents and the problems in dealing with acute side effects and complications especially in young patients. Simultaneous CTX has proven to be a significant factor for the occurrence of acute high-grade adverse events and for interruptions of radiotherapy.

## 1. Introduction

Primary central nervous system (CNS) tumors in children require a multimodal therapeutic approach, which includes tumor resection with/without adjuvant radiotherapy and CTx. The choice of treatment modalities is based on neuropathology diagnosis and specific treatment protocols. Proton therapy has emerged as the preferred alternative to classical photon beam radiation due to its precise targeting and ability to deliver higher doses to the tumor while sparing surrounding healthy tissue [1,2].

This has led to an increased interest in optimizing multimodal treatment options for pediatric CNS tumors, particularly focusing on the benefits of proton therapy. Recent studies have predominantly examined the outcomes and long-term toxicities associated with proton beam therapy (PBT), both with and without concurrent CTx [3,4].

Although it is well-documented that simultaneous radiochemotherapy is associated with a higher incidence of high-grade adverse events, there is a scarcity of studies investigating acute toxicities during the radiation period and their impact on the feasibility of proton therapy with concurrent CTx [5,6,7].

Factors that may affect the feasibility of proton therapy and simultaneous CTx often arise before irradiation and include burden from prior treatments such as surgery or CTx, VP shunt dysfunction or infection, post-surgical complications such as posterior fossa syndrome, nerve damage, and long-term toxicities from previous CTx, such as neuropathies and nephropathy.

During the treatment period, careful attention must be given to acute toxicities arising from radiation and concurrent CTx, as these toxicities may necessitate dose reductions or interruptions, complicating treatment protocols. It is equally important to document and manage side effects, whether through unplanned hospitalizations or minor or major medical interventions.

The primary objective of this retrospective cohort study was to identify the adverse events (AEs), including their frequency, type and severity, that occurred during proton therapy with concomitant CTx compared to therapy without simultaneous CTx in patients with primary pediatric CNS tumors. The main secondary objective was to determine the impact of AEs on the feasibility of this combined treatment approach.

## 2. Patients and Methods

### 2.1. Eligibility Criteria

This study included patients < 18 years of age with a primary CNS tumor who underwent proton beam therapy at the West German Proton Center between September 2013 and February 2017 with or without concurrent chemotherapy. The chemotherapy was administered and monitored by the Pediatric Hematology and Oncology Department of the University Hospital of Essen. The patients originate either from the Department of Pediatric Hematology and Oncology, Pediatrics III, University Hospital of Essen, Germany, or from other national and international centers for pediatric hematology and oncology. All patients were registered in the KiProReg (Registry study of Standard Proton Therapy in Children at West German Proton Therapy Center, University Hospital of Essen; German Clinical Trials Register: DRKS00005363).

### 2.2. Study Design and Data Collection

The study was conducted as a monocentric, retrospective, non-randomized, and open-label study. The primary endpoint of the study was the assessment of AEs (safety), including their frequency, type and severity occurring during all PBT courses (from start to finish, not during the posttreatment period) as well as their relationship to the irradiated anatomical sites, simultaneous chemotherapy, and other clinical cofactors (age, VP shunt, tumor entity, and prior high-dose or intrathecal chemotherapy). Secondary endpoint was the feasibility analysis including the assessment of PBT interruptions (frequency, duration, reasons, relationship to clinical cofactors), interruption of simultaneous chemotherapy in sPBCT courses (proportion of applied planned chemotherapy, reasons, and relationship to clinical cofactors), unplanned hospitalizations (frequency, duration, reasons) and supportive therapy (type, and duration of application).

For this retrospective study, data was collected out of medical records. This basic information contained age at radiation start, gender, date of diagnosis, entity and location of malignancy, former therapies including radiotherapy and CTx, such as surgeries or specific pre-existing conditions.

The collected data comprise a total of 200 radiotherapy courses from 199 patients. The analysis is based on the individual radiotherapy courses.

Data dealing with treatment information during the period of PBT was taken from patient records updated every seven to fourteen days or in case of acute consultations. This data contained dose and target volumes, distinguishing between radiation field of the primary brain tumor, craniospinal axis as well as radiologically documented metastases.

The prescribed radiation dose was specified in Gray (Gy). A fixed relative biological effectiveness (RBE) value of 1.1, referenced to Co-60, was used across all treatment plans. Consequently, proton doses are presented as Gy (RBE), derived by multiplying the physical proton dose by 1.1. Proton beam therapy was delivered either with uniform scanning or pencil-beam scanning for focal or boost treatments.

If CTx was administrated during the time of radiation drug substances and applied doses were documented with percent reduction according to the particular regime. Three different simultaneous chemotherapy regimens were used: two less intensive chemotherapy regimens in the form of (i) weekly intravenous vincristine (planned six to seven applications during an sPBCT course) or (ii) daily oral administration of temozolomide (7 days per week) and (iii) one intensive chemotherapy regimen which was administered as multi-day intravenous courses with two or three cytotoxic agents per course (vincristine, cyclophosphamide or ifosfamide, carboplatin/cisplatin, etoposide, planned on a case-by-case basis with two to three courses during the sPBCT course at intervals of two to three weeks). It was assumed that the expected myelosuppression would be more pronounced with the intensive intravenous therapies than with the oral therapy using temozolomide or the weekly intravenous administration of vincristine. Chemotherapy interruptions either followed protocol-specified rules or local practice depending on the underlying cause.

Delays of therapy, whether it affected radiation or CTx administration, were taken note of by addressing the amount of radiation delay or reduced CTx dose as well as the causative high-grade adverse event (HGAE) and the time point during radiation course. CTx interruptions were defined as either delayed courses or missed doses of weekly or daily oral cytostatic agents. Regarding radiotherapy, a major delay was defined as an interruption of radiotherapy for at least three days, which could impact the effectiveness of the treatment.

If children were hospitalized during the treatment due to toxicity events, days of hospitalization and causing event(s) were recorded separately. To ensure that every high-grade AE possibly influencing the therapy is recognized, we documented every HGAE in total, regardless to treatment delays or hospitalizations.

An infection was defined as either a microbiologically, clinically or radiologically documented infection or as fever with or without neutropenia resulting in hospitalization and intravenous application of antibiotics.

Depending on the different agents, the administration of supportive care followed standardized institutional guidelines and was documented by the treating physicians. Transfusions of red blood cells or platelets, as well as the administration of G-CSF, were consistently ordered according to established international guidelines (hemoglobin < 7 g/dL; platelets < 20,000/µL in the absence of bleeding; G-CSF for absolute neutrophil counts < 1000/µL).

### 2.3. Ethical Approval

The patients, where appropriate, and their legal guardians gave their written informed consent prior to enrollment in the prospective registry study KiProReg. The approval for this registry study has been granted by the local ethical committee of the University Hospital of Essen (16-7261-BO).

### 2.4. Safety Analysis

Toxicity was graded according to Common Terminology Criteria for Adverse Events (CTCAE), Version 4.03. The degree of toxicity was assessed and recorded by the attending pediatrician during weekly assessments. CTCAE grade 1 and 2 were defined as mild and moderate AEs and CTCAE grade ≥ 3 as high-grade adverse events (HGAEs) independently of their seriousness (CTCAE grade 3, 4 or 5), their outcome (hospitalization, permanent damage or death) or their expectedness (expected or unexpected).

### 2.5. Statistical Analysis

SPSS Statistics version 29 (IBM Corp. Released 2022. IBM SPSS Statistics for Mac, Version 29.0. Armonk, NY, USA: IBM Corp) was employed for data management and statistical analyses. The distribution and relationships of the attributes were assessed and compared using cross-tabulations and binary logistic regression. Statistical significance was defined for a *p*-value ≤ 0.05.

## 3. Results

### 3.1. Patient Clinical Characteristics

A total of 199 children and young adolescents, encompassing 200 proton beam therapy (PBT) courses were enrolled in this retrospective study.

Among these patients, 121 received radiation therapy at the time of their initial diagnosis, while 79 underwent PBT at recurrence or progression. The median age at the start of PBT was 7.4 years, with an age range of 0.9 to 17.9 years. This cohort included 116 male patients (117 PBT courses as one patient received two cycles in total), and 83 female patients (83 PBT courses) (Table 1).

**Figure 1:**
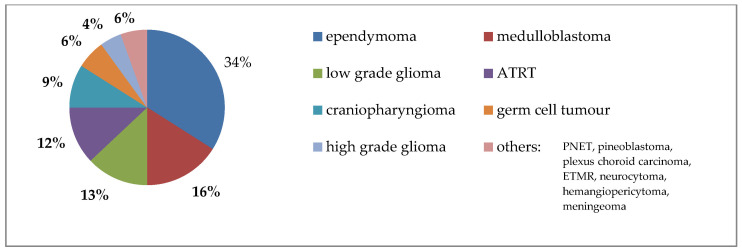
Distribution of tumor entities sorted by courses: about half of irradiated tumors were ependymomas and medulloblastomas (ATRT—atypical teratoid rhabdoid tumor, PNET—primitive neuroectodermal tumor, ETMR—embryonal tumor with multilayered rosettes).

The entities included in this study comprised primary CNS tumors localized in the brain or spinal cord. The most prevalent entities were ependymomas (*n* = 68), medulloblastomas (*n* = 32), low-grade gliomas (*n* = 26), atypical teratoid rhabdoid tumors (ATRT, *n* = 24), craniopharyngiomas (*n* = 18), and germ cell tumors (*n* = 12) (Figure 1). CNS tumors were classified according to the current World Health Organization (WHO) grading system: CNS WHO grade 1 (*n* = 43), grade 2 (*n* = 22), grade 3 (*n* = 60), and grade 4 (*n* = 62). Additionally, 12 patients had tumors being not graded in the CNS WHO grading system (6 germinomas and 6 non-germinomatous tumors). Due to the specialization of national treatment centers, the distribution of entities may not reflect their distribution in the general population.

At the start of PBT, 137 patients had a central venous device, 39 children had a ventriculoperitoneal (VP) shunt, and 37 had a Rickham or Ommaya Reservoir. To ensure proper nutrition during radiation therapy, some patients had a percutaneous endoscopic gastrostomy (PEG, *n* = 16) or a nasogastric tube (*n* = 10). Two patients had a suprapubic catheter. Due to their age (mostly under 6 years) or their limited general condition, half of the PBT courses were conducted under intravenous analgesia with propofol.

Prior to the start of PBT, 101 children had received conventional CTx either at initial diagnosis (*n* = 81) or at relapse (*n* = 20). High-dose CTx was administered in 12 cases, and intrathecal CTx in 36 cases. Despite the shared disease entity, treatment plans and regimens frequently differed, in part due to the international origin of the patients.

In 190 cases, surgery (tumor resection or biopsy) was performed prior to the start of PBT, i.e., in 113 patients as part of their first-line therapy and in 77 patients at recurrence. Radiation therapy with photons or protons in an overlapping area prior to PBT was performed in six cases, either at initial diagnosis (*n* = 4) or at relapse (*n* = 2). One patient who underwent re-irradiation received PBT twice at the West German Proton Center and was counted twice in this study, once for each radiation course.

At staging prior to PBT 179 patients showed local disease only (M0 stage), eight had tumor cells in the cerebrospinal fluid (CSF) (M1 stage), three had metastases in the neurocranium (M2 stage), and 10 had spinal metastases (M3 stage). A total of 62 patients received radiation while being in complete remission, 85 in partial remission, 18 had stable disease, and 35 experienced disease progression prior to the start of PBT.

Due to prior therapy or the underlying tumor disease itself, certain patients began proton therapy with high-grade neurological disabilities (*n* = 23) or panhypopituitarism (*n* = 22).

### 3.2. Current Radiotherapy

A total of 199 patients received 200 irradiation courses (median PBT dose: 54 Gy (RBE), range: 24–74 Gy (RBE), fraction dose: 1–4 Gy (RBE), median: 1.8 Gy (RBE)). The main irradiation field included 106 infratentorial tumor sites, nine spinal tumor sites, and 85 supratentorial tumor sites with 36 tumors of them located in the intra- or suprasellar regions. Radiation of solid metastases was performed additionally to primary tumor bed radiotherapy or separately in eight cases (median PBT dose: 49.4 Gy (RBE)). Craniospinal irradiation was administered to 38 children (median PBT dose: 23.4 Gy (RBE), range: 18.0–36.0 Gy (RBE), primary tumor site: 31/38 infratentorial, 7/38 supratentorial, 1/38 spinal). Since the main objective of this analysis was to assess the feasibility of simultaneous PBCT, no more detailed radiation analysis was performed.

### 3.3. Simultaneous Chemotherapy

CTx was administrated concurrently in 52 courses of radiotherapy according to various national study protocols. Twenty-five patients received weekly intravenous Vincristine injections (medulloblastoma *n* = 15, ependymoma *n* = 10, median 6 application, range 1–8). Twelve patients with atypical teratoid rhabdoid tumors (ATRT) and one patient with a PNET underwent one or two courses (median 2) of intensive intravenous CTx according to the EURHAB Registry or other local protocols including vincristine, ifosfamide, cyclophosphamide, carboplatin, cisplatin and etoposide with two or three planned cycles per radiation course. Simultaneous oral CTx with temozolomide (4 to 7 days per week) was applied in 14 cases [high grade glioma (HGG) *n* = 8, medulloblastoma *n* = 5, embryonal tumor with multilayered rosettes (ETMR) *n* = 1, median 45 applications, range 30–49].

### 3.4. Adverse Events During Radiotherapy with Respect to Anatomical Site

Over the course of 200 irradiation courses with or without sCTx, 194 patients (97%) experienced at least one adverse event. A total of 704 adverse events (mean 3.4 per course) were recorded, including 617 toxicities graded as CTCAE grade 1 or 2 (87.6%) and 87 toxicities graded as CTCAE grade 3 or 4 (12.3% of all HGAEs occurring in 67 PBT/sPBCT courses, Table 2).

The most common adverse events, regardless of the CTCAE grade, were skin and subcutaneous disorders (*n* = 149) and blood and hematopoietic system disorders (*n* = 149), followed by infections (*n* = 77), nausea (*n* = 55), and vomiting (*n* = 54). The most common high grade adverse events (CTCAE grade 3 or 4) were blood and hematopoietic system disorders (*n* = 43), infections (*n* = 19), hyponatremia (*n* = 5), vomiting (*n* = 5), and various other conditions (*n* = 21).

High-grade hematotoxicity (CTCAE grade 3 or 4) was observed primarily in patients receiving CSI (*n* = 22, 57.9% of CSI patients, primary tumor site: 4/22 supratentorial, 18/22 infratentorial) but also in patients treated with local radiotherapy only (infratentorial tumor site *n* = 16, supratentorial tumor site *n* = 6, and spinal tumor *n* = 1). Infections were documented in 18 cases, mainly in patients with infratentorial tumors (*n* = 11, without CSI), CSI (*n* = 4, primary tumor site: 3/4 infratentorial, 1/4 supratentorial), and supratentorial tumors (*n* = 3, without CSI). Vomiting and hyponatremia each occurred in five cases, with vomiting particularly observed in patients with infratentorial tumors (*n* = 3) and other patients with CSI (*n* = 2), and hyponatremia in patients with intrasellar tumors (*n* = 4) and CSI (*n* = 1). A fatal HGAE (CTCAE° 5) was not observed.

### 3.5. High Grade Adverse Events with Respect to Simultaneous Chemotherapy

A total of 33 out of 52 patients (63.5%) who received simultaneous CTx experienced 42 HGAEs (mean 1.2 HGAE per patient). In contrast, in patients treated with PBT only 45 HGAEs occurred in 34 out of 148 patients (23%, mean 1.4 HGAE per patient). This difference was statistically significant (*p* < 0.001).

The HGAEs in patients with sPBCT were primarily hematotoxicity (*n* = 26, mainly neutropenia), infections (*n* = 7), and peripheral neurotoxicity (*n* = 3).

The potential risk factors for the occurrence of HGAEs [age class < 5 years), sex (male), ATRT histology (yes), relapse prior PBT (yes), presence of a VP shunt (yes), metastatic status prior to PBT (yes), disease status prior PBT (SD or PD), HD-CTx prior PBT (yes), intrathecal CTx prior PBT (yes), simultaneous CTx to PBT (yes), craniospinal irradiation (yes), were analyzed in a binary logistic regression model. This model yielded odds ratios > 1 and a significant association with the occurrence of HGAE for ATRT histology, presence of VP-shunt, HD-CTx prior PBT, CSI and simultaneous CTx (Table 3).

### 3.6. Interruption of Radiotherapy

Temporary interruptions of PBT/sPBCT due to toxicity or non-compliance occurred during 19 radiation courses, either once or multiple times (Table 4). A total of 26 interruptions (total cumulative days = 70; median one day per patient, range 1–19 days per patient) occurred. The causes for these temporary interruptions were mainly VP shunt dysfunctions (*n* = 6; total cumulative days = 32, median days per patient 5 days, range 3–19 days per patient) and infections (*n* = 11; total cumulative days = 28, median one day per patient, range 1–14 days per patient). However, major delays occurred in only five cases, also due to VP shunt dysfunction (for 19, 7 and 3 days) and infections (for 14 and 4 days), respectively. These unplanned PBT interruptions did not affect the planned biological effective radiation dose, as the missed fractions were compensated for by extending the overall treatment time. This was done on a case-by-case basis by adjusting the prescribed single and total doses and, if necessary, of the size of the irradiated volume at the discretion of the radiation oncologist.

Seven patients out of the 52 patients (13.5%) who received sPBCT had to interrupt their radiation treatment due to HGAEs. In contrast, only 12 patients out of the 148 patients (8.1%) who received PBT without any simultaneous chemotherapy experienced a HGAE associated treatment interruption (*p* = 0.201).

Potential risk factors for the occurrence of interruptions of PBT [age class (<5 years), sex (male), relapse prior to PBT (yes), presence of a VP shunt (yes), metastatic status prior to PBT (yes), disease status prior to PBT (SD or PD), HD CTx prior to PBT (yes), intrathecal prior to PBT (yes), ATRT histology (yes), simultaneous CTx to PBT (yes), craniospinal irradiation (yes), occurrence of HGAE (yes), occurrence of infection during PBT (yes)] were analyzed in a binary logistic regression model. This model yielded odds ratios > 1 and a significant association with the occurrence of one or more radiotherapy interruptions in the same PBT courses only for the factors occurrence of HGAE and occurrence of infection (Table 5).

### 3.7. Interruption of Simultaneous Chemotherapy

**Figure 2:**
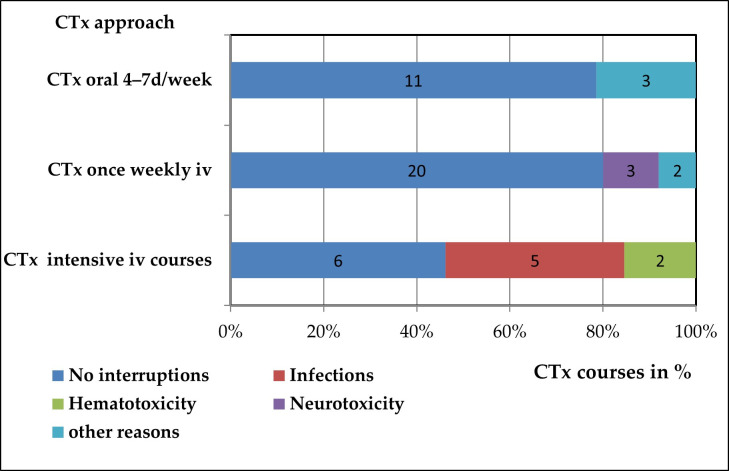
Chemotherapy interruptions dependent on CTx approach and kind of toxicity. There was a significantly higher rate of CTx interruptions in sPBCT with intensive intravenous CTx courses in comparison to oral and weekly CTx regimens (*p* < 0.001). The numbers in the bars represent the number of patients. (iv = intravenous(ly), CTx = chemotherapy).

Fifteen (28.8%) out of the 52 patients receiving sPBCT experienced unplanned delays or interruptions in their CTx regimen, regardless of the route of administration (Figure 2).

Among the children receiving intravenous intensive CTx courses (*n* = 13, 12 of them with ATRT), seven did not complete the originally planned number of courses, resulting cumulatively in only 17 out of 24 planned courses (70.8%) being administered. The interruptions were necessary due to high grade infection (*n* = 5) and hematotoxicity (*n* = 2).

Twenty-five patients received CTx with Vincristine on a weekly basis. The administration of Vincristine was canceled six times in five patients due to peripheral neuropathy (*n* = 3) and other minor reasons (constipation, allergy, each *n* = 1) resulting cumulatively in 148 administered of 154 planned applications (96.1%).

Patients receiving oral CTx (*n* = 14) experienced interruptions in three cases due to elevated liver transaminases or thrombocytopenia. In this group cumulatively 544 days out of 580 planned CTx days (93.7% of planned applications) were administered.

In summary, the rate of actually applied planned CTx doses was significantly lower in patients with intensive intravenous CTx courses than in those with the less intensive daily oral and weekly intravenous CTx (*p* < 0.001).

In a binary logistic regression model including potential risk factors for CTx interruptions [occurrence of HGAE (yes), occurrence of infection during PBT (yes)] only the occurrence of infection (Odds ratio 3.905, 95% confidence interval 1.005 to 15.174, *p* = 0.049) was significantly associated with CTx interruption. The additional inclusion of other potential risk factors in the model, as in the model for PBT interruptions, did not result in any significance for any factor.

### 3.8. Unplanned Hospitalizations

In 33 of 200 (16.5%) PBT courses, children underwent singular or multiple unplanned hospitalizations. A total of 50 unplanned hospitalizations, accounting for 355 cumulative hospitalization days, were documented (median per patient 7 days; range 1–58 days).

Children were hospitalized 33 times due to infections (cumulative hospitalization days 211, median per patient 5 days, range 1–44 days), seven times due to electrolyte derailments (cumulative hospitalization days 39 days, median per patient 4 days, range 2–13 days), six times due to VP shunt dysfunction (cumulative hospitalization days 76 days, median per patient 9 days, range 2–29 days) and four times due to neurotoxicity, such as seizures and motoric dysfunctions (cumulative hospitalization days 11 days).

Unplanned hospitalization tended to be more often necessary in patients receiving sPBCT (13/52 patients, 25%) than in patients undergoing PBT only (20/148 patients, 13.5%) (*p* = 0.11). When comparing all different subgroups, hospitalization associated with HGAE was more frequently required in patients receiving intensive intravenous CTx therapy than in patients receiving weekly intravenous (*p* = 0.053) or oral CTx therapy (*p* = 0.018) or in patients receiving only PBT (*p* < 0.001).

### 3.9. Supportive Therapy

Antiedematous therapy with dexamethasone was required in 25 cases (12.5%, prophylactically in 7.5%, symptom-dependent in case of clinical signs of an increased intracranial pressure in 5%), with durations ranging from 2 to 51 days (median 28 days, cumulatively 631 days, median dose 0.1 mg/kg/day, range 0.01–0.21 mg/kg/day). Antiemetics were administered in 73 patients (36.5%, permanently in 23.5%, symptom-dependent in case of vomiting and/or nausea 13.0%).

Antibiotics were applied in 32 cases (16%) due to confirmed or suspected infections cumulatively for 371 days of intravenous administration (median days per patient 8, range 3–35 days) and cumulatively for 151 days of oral administration (median days per patient 7, range 4–17 days). Intravenous antifungals were used in 2 cases for duration of 8 and 9 days, respectively.

Children with blood and hematopoietic disorders were treated with platelet concentrates, erythrocyte concentrates and granulocyte-colony stimulating factor (G-CSF). One patient developed a high-grade thrombocytopenia requiring two platelet concentrate transfusions. Erythrocyte concentrate transfusions due to high grade anemia were necessary in three cases, with a total of five blood bags used. G-CSF for neutropenia was administered in 15 cases (cumulatively on 130 days, median days per patient 10, range 2–14 days).

Pain-relieving oral analgesics were applied in 37 cases (18.5%), with 29.7% of these prescriptions being permanent during the time of PBT. High grade treatment-associated pain required intravenous analgesics in four cases.

Total parenteral nutrition was administered in two cases for a total of 35 and 4 days, respectively. Digestive agents were used in 52 cases (26%), with 29 cases requiring permanent administration.

## 4. Discussion

Proton therapy is becoming the new standard for the radiotherapy of solid pediatric CNS tumors in industrialized countries, demonstrating potential advantages over conventional photon therapy, i.e., such as the potential for reduced long-term toxicities while maintaining equivalent or improved tumor control [4,9,10,11,12]. Moreover, Vazquez et al. (2023) reported that PBT is particularly advantageous for young patients due to its precision, which spares healthy brain parenchyma tissues and reduces long-term toxicity. Additional research supports these findings, indicating improved short-term progression-free (PFS) and overall survival (OS) with PBT in CNS tumors of children, adolescents and young adults. However, there is a lack of randomized prospective multicenter trials in larger cohorts in the same tumor entity evaluating survival outcome as well as acute and long-term toxicities. As reported for photon therapy metastatic and recurrent diseases remain the poorest prognostic factors which detrimentally affect the clinical outcome [13,14,15].

While the most recent published studies deal with the outcome or long-term side effects of PBT, the aim of our study was to investigate the feasibility of PBT with a particular focus on the feasibility and safety of a simultaneous radiochemotherapy (sPBCT). In our cohort, 97% of patients experienced at least one and in mean three adverse events which were mostly graded as mild or moderate. High-grade adverse events (CTCAE grade 3 or 4) were documented in one third of PBT/PBSCT courses and were in particular hematotoxicity (especially prolonged and deep neutropenia) and infections, and more rarely neurotoxicity. Occurrence of HGAEs, interruptions of PBT and need of hospitalization was significantly more frequent in patients with sPBCT and especially in patients receiving simultaneous intensive intravenous chemotherapy. In particular, younger patients with ATRT have borne the highest risk for the occurrence of HGAEs (especially infections) and therapy interruptions. This confirmed the results reported by Owusu-Agyemang et al. (2025) who used comparable to us propofol-based total intravenous anesthesia in young children (median age 4.7 years, *n* = 194) with different CNS tumor for accurate delivery of PBT and observed a higher rate of therapy interruptions in this age group. Compared to the older age group (median 12.2 years, *n* = 267) without anesthesia, the younger age group showed an increased risk for HGAE-related emergency room visits and unplanned hospital stays, without this affecting overall survival [16]. However, to assess the impact of sPBCT and its intensity on acute and long-term toxicity and overall survival compared to current high-dose chemotherapy regimens, larger cohort studies, particularly in patients with ATRT, are needed.

The distribution of toxicities shows notable similarities to the findings reported by Nguyen et al., who also observed a high incidence of skin toxicity, nausea and vomiting, and hematological toxicity among a cohort of 272 medulloblastoma patients undergoing craniospinal irradiation, as well as mentioned in other studies [17,18,19,20,21,22]. Skin and subcutaneous disorders are the most prevalent toxicities observed in our patient group undergoing proton therapy, regardless of grade. However, it is noteworthy that high-grade cases of radiation dermatitis are absent, suggesting that this condition does not generally compromise the feasibility of PBT or sPBCT. Recent studies have highlighted the prevalence and management of radiation dermatitis in pediatric oncology patients treated with proton therapy. A study on PBT for pediatric non-central nervous system malignancies showed that while skin reactions are common, the grade is usually mild to moderate, and high-grade dermatitis is rare [23].

Hematotoxicity was the most common high grade adverse event among patients receiving concurrent CTx and is a well-documented risk in pediatric cancer patients undergoing combined modality treatment. CTx agents such as cyclophosphamide, ifosfamide, cisplatin, carboplatin and vincristine are known to cause high grade myelosuppression, which is exacerbated when combined with radiation therapy. A study by Mizumoto et al. (2019) on the combined use of proton therapy and CTx in pediatric patients found a significant incidence of hematologic toxicities, including neutropenia and thrombocytopenia, which aligns with the findings in this study [24]. In our study, most HGAEs were hematotoxicity with high-grade leukopenia and neutropenia, with more than half of the affected patients having received craniospinal irradiation. This finding is consistent with results of other studies that used photons or protons and have already reported an increased incidence of high grade hematotoxicity without a relevant increased risk of CSI-associated infections [17,20,21,22,25]. Infections were the second most frequent HGAEs in our cohort and increased the risk for PBT and CTx interruptions. Although this association reached statistical significance by a narrow margin (*p* = 0.049), the observed Odds Ratio of >3 suggests considerable clinical relevance, meaning that patients with an infection are three times more likely to face treatment interruptions. They occurred less frequently in association with CSI, rather with simultaneous intensive chemotherapy and as device complication. Whether CSI with PBT compared to CSI with photons can reduce the rate of acute high-grade hematological toxicity and high-grade infections through radiation-sparing techniques is currently unclear and requires further investigation in larger retrospective and prospective cohorts.

Hyponatremia as a high-grade adverse event was rare but mostly seen in patients with intrasellar tumors (*n* = 4/5), though less frequent, it has been documented in the context of hypothalamic-pituitary axis radiation, particularly in tumors involving the sellar region. Close electrolyte monitoring during radiation of sellar-region tumors are essential [26].

Recent studies in adult brain tumors comparing photon therapy to PBT have already shown a reduction in acute side effects [27]. In the majority of cases in our study, toxicity-related interruptions of proton therapy were limited to one or two consecutive days, and the total number of interruptions remained low across the 200-PBT-course cohort. This favorable outcome could also be associated with efficient clinical management, allowing timely resumption of radiotherapy despite concurrent treatment of complications such as infections or neurotoxicity. Major interruptions of PBT due to toxicity resulted in treatment delays of more than three days in only a few cases. However, when such prolonged interruptions did occur, they were mostly associated with VP shunt malfunction or high-grade infections.

Considering the feasibility of sPBCT in our study, 28.8% of the patients with simultaneous CTx did not receive the full number of originally planned courses, in particular in sPBCT with intensive intravenous CTx. Interruptions were predominantly due to infections and hematologic toxicity, and in line with findings reported in the existing literature [28]. The known side effects of CTx may be potentiated by concurrent irradiation during this period. A study comparing the incidence of infections during CTx alone with that during concurrent CTx and radiation would be valuable in this context. Patients receiving weekly CTx, such as Vincristine, one or more administrations were canceled 20% of the time due to neurotoxicity and constipation. Neurotoxicity is a well-known side effect of Vincristine, manifesting as peripheral neuropathy, motor weakness, and other neurological symptoms. The literature indicates that these neurotoxic effects not only impair the quality of life of patients but also lead to treatment delays [29]. Hess et al. (2018) reported and in our study it was confirmed that concurrent CTx with PBT is feasible and can be well-tolerated, provided that careful monitoring is implemented. However, the potential for increased toxicity, as observed in this study, necessitates the optimization of combined treatment protocols [30]. Further research is needed to develop preventive strategies to minimize these side effects and ensure the continuous and not interrupted administration of planned sPBCT in order to achieve the best possible treatment and survival outcome in children with cancer.

While previous studies have addressed treatment-related toxicities, they often lack detailed insights into clinical management and the resulting hospital admissions [20]. Current data on unplanned hospitalizations during proton therapy in pediatric patients reveal notable clinical challenges. At least one sixth of patients required at least one unplanned hospital stay. It is notable that causes of hospitalization (infections, electrolyte imbalances, shunt dysfunctions, and neurotoxic symptoms) often match with the reason of treatment interruptions, but frequency and duration of hospitalizations far exceed duration of treatment interruptions, due to improved clinical management by experienced interdisciplinary teams. Although electrolyte disturbances are not a primary reason for interrupting therapy, they frequently needed inpatient care due to their severity and the need for complex clinical and electrolyte monitoring. These complications are common in tumors of the sellar region and may be exacerbated by intensive treatment regimens [31].

Although proton therapy offers clear advantages in sparing healthy tissue and reducing long-term toxicities compared to conventional photon therapy, our findings emphasize the ongoing need for comprehensive supportive care during treatment. The frequent use of corticosteroid support reflects the complex clinical management required to treat and avoid tumor- and therapy-induced cerebral edema [32]. Especially patients receiving craniospinal irradiation with blood and hematopoietic disorders required various interventions, such as platelet concentrates, erythrocyte concentrates, and granulocyte-colony stimulating factor (G-CSF), indicating the critical need for hematopoietic support to prevent high grade treatment interruptions and especially infectious complications. Total parenteral nutrition highlights the need for comprehensive nutritional and gastrointestinal support to ensure patient well-being and treatment continuity, especially in young children.

In conclusion, the importance of supportive therapy during radiation and also proton therapy cannot be overstated. Overall, these results reinforce the importance of a multidisciplinary approach to managing acute toxicities during PBT and sPBCT. Future research should focus on optimizing radiotherapy and CTx protocols as well as supportive care and investigating the differential impact of concurrent CTx to further improve treatment tolerability and outcomes in pediatric CNS tumor patients.

The limitations of this study lie in the small cohort size with regard to the different tumor entities and the number, different types and intensity of simultaneous CTx, the single-center and retrospective study design, and the lack of a control cohort for photon radiotherapy. In addition, future studies with larger cohorts should explore the use of propensity score matching or weighting to reduce confounding variables and improve covariate balance and the internal validity of the analysis. Furthermore, it should be noted that in our binary logistic regression models bias from multiple testing with a potential type I error explosion cannot be ruled out due to heterogeneous patient cohorts and a low events-per-variable ratio. This may lead to an overestimation of effect sizes, for example in our study regarding the occurrence of infection as a reason for treatment interruptions.

While a prospective, randomized, controlled multicenter trial would be the best way to compare proton and photon radiotherapy with the same type of sPBCT, such a study design does not appear ethically feasible given that proton radiotherapy has become the new standard of care in industrialized countries for reducing late events, particularly in younger patients. Multicenter radiotherapy registries and multicenter, multinational meta-analyses could offer alternatives for analyzing large cohorts with regard to short- and long-term toxicity and survival rates [13,14,17].

## 5. Conclusions

This study underscores the feasibility of proton therapy, both with and without concurrent CTx, in the treatment of pediatric CNS tumors, managing especially the acute side effects. When PBT is combined with CTx, management can be challenging due to the potential for acute toxicities. By identifying and analyzing these potential risks and their impact, future treatment protocols can be adapted to provide the most effective therapy for pediatric CNS tumors with the lowest possible and acceptable side effects.

Supportive therapies are critical in the management of acute toxicities in pediatric CNS tumors in order to maintain treatment continuity. The significant incidence of unplanned hospitalizations due to high grade side effects further emphasizes the importance of proactive and comprehensive supportive care strategies. These interventions are vital for minimizing treatment interruptions and optimizing therapeutic outcomes for pediatric patients undergoing proton therapy.

The need for personalized treatment plans is evident, considering the diverse radiation doses and target sites required to address the unique characteristics of each patient’s conditions. Additionally, the integration of multimodal therapies, including concurrent CTx, must be carefully managed to balance efficacy with the risk of increased toxicities. This study underlines the need for an experienced neuro-oncological center of maximum care in which pediatricians, radiotherapists, anesthesiologists, neuroradiologist, intensive care physicians and neurosurgeons work closely together on an interdisciplinary basis to enable radiotherapy with as few complications as possible.

Future research should focus on optimizing combined treatment protocols and supportive care strategies to enhance the overall effectiveness of proton therapy while mitigating adverse effects.

## Figures and Tables

**Table 1:** Patient clinical characteristics (*n* = 199 patients, *n* = 200 PBT/sPBCT courses).

Characteristics	All Patients (%/Range)	sPBCT (%/Range)	PBT Only (%/Range)
All PBT courses *	200 * (100%)	52 (26%)	148 (74%)
gender	male 117 (58.5%)	male 34 (65.4%)	male 83 (56.1%)
	female 83 (41.5%)	female 18 (34.6%)	female 65 (43.9%)
age at initial diagnosis	median 6.3 years (0.04–17.5)	median 6.7 years (1.0–16.3)	median 6.2 years (0.04–17.5)
age at start of proton therapy	median 7.4 years	median 7.1 years	median 7.5 years
	(0.9–17.9)	(1.4–16.4)	(0.9–17.9)
Tumor site at PBT			
supratentorial	85 (42.5%)	15 (28.8%)	70 (47.3%)
infratentorial	106 (53.0%)	35 (67.3%)	71 (48.0%)
spinal	9 (4.5%)	2 (3.8%)	7 (4.7%)
CNS WHO grade			
1	43 (21.5%)	0 (0%)	43 (29.1%)
2	22 (11.0%)	2 (3.8%)	20 (13.5%)
3	61 (30.5%)	15 (28.8%)	46 (31.1%)
4	62 (31.0%)	35 (67.3%)	27 (18.2%)
Metastatic stage prior to PBT/sPBCT			
not applicable	12 (6.0%)	0 (0%)	12 (8.1%)
M0	179 (89.5%)	47 (90.4%)	132 (89.2%)
M1	8 (4.0%)	4 (7.7%)	4 (2.7%)
M2	3 (1.5%)	0 (0%)	3 (2.0%)
M3	10 (5.0%)	1 (1.9%)	9 (6.1%)
Devices installed prior PBT/sPBCT			
central venous catheter	137 (68.5%)	43 (82.7%)	94 (63.5%)
Ommaya/Rickham reservoir	37 (18.5%)	11 (21.2%)	26 (17.6%)
VP shunt	39 (19.5%)	9 (17.3%)	30 (20.3%)
PEG/nasogastric tube	26 (13.0%)	6 (11.5%)	20 (13.5%)
Suprapubic catheter	2 (1.0%)	1 (1.9%)	1 (0.7%)
Response evaluation criteria prior to PBT/sPBCT			
CR	62 (31.0%)	19 (36.5%)	43 (29.1%)
PR	85 (42.5%)	25 (48.1%)	60 (40.5%)
SD	18 (9.0%)	5 (9.6%)	13 (8.8%)
PD	35 (17.5%)	3 (5.8%)	32 (21.6%)
Timing of PBT/sPBCT			
at primary diagnosis	121 (60.5%)	44 (84.6%)	77 (52.0%)
at recurrence/progression	79 (39.5%)	8 (15.4%)	71 (48.0%)
Treatment prior to PBT/sPBCT			
prior tumor surgery	190 (95.0%)	52 (100%)	138 (93.2%)
(at initial diagnosis/at recurrence)	145/45	49/3	96/42
prior photon radiotherapy	4 (2.0%)	1 (1.9%)	3 (2.0%)
in an overlapping area			
prior proton beam therapy	2 (1.0%)	0 (0%)	2 (1.3%)
in an overlapping area			
prior CTx	101 (50.5%)	26 (50.0%)	75 (50.7%)
(at initial diagnosis/at recurrence)	81/20	23/3	58/17
prior HD CTx	12 (6.0%)	0 (0%)	12 (8.1%)
prior intrathecal CTx	36 (18.0%)	14 (26.9%)	22 (14.9%)

* One patient received twice PBT (anaplastic ependymoma of the posterior fossa), each as first-line and second-line therapy in an 8.5-month interval due to a recurrence, and were counted twice. He did not receive any simultaneous chemotherapy and showed no high-grade AE in either of his two PBT courses. Abbreviations: PBT = proton beam therapy; sPBCT = proton beam therapy with simultaneous chemotherapy; CNS = central nervous system, WHO = World Health Organization; VP = ventriculoperitoneal; PEG = percutaneous endoscopic gastrostomy; HD = high dose, CR = complete remission, PR = partial response, SD = stable disease, PD = progressive disease (defined according to McDonald criteria) [8].

**Table 2:** High-grade adverse events (CTCAE grade 3 or 4, *n* = 87) with respect to tumor site, CSI and simultaneous radiochemotherapy (sPBCT).

Radiation Field	Supra-	Intrasellar/	Infra-	Spinal	CSI	Overall
[Number of Courses]	Tentorial ^#^	Suprasellar	Tentorial			Courses
(with sPBCT)]	*n* = 43 *	*n* = 35 *	*n* = 76 *	*n* = 8 *	*n* = 38	*n* = 200
Blood and hematopoietic system disorders	6 (5)	-	14 (11)	1 (1)	22 (9)	43 (26)
Infections and infestations	3 (2)	-	12 (3)	-	4 (2)	19 (8)
Vomiting	-	-	3 (1)	-	2 (0)	5 (1)
Electrolyte imbalance	-	4 (0)	-	-	1 (0)	5 (0)
Central neurotoxicity	1 (1)	1 (0)	2 (0)	-	-	4 (0)
Constipation	1 (1)	1 (1)	-	1 (0)	-	3 (2)
Peripheral neurotoxicity	2 (2)	-	1 (1)	-	-	3 (3)
Nausea	-	-	2 (0)	-	-	2 (0)
Skin disorders	-	-	-	-	1 (0)	1 (0)
Diarrhea	-	-	-	-	1 (1)	1 (1)
Hepatobiliary disorders	-	-	1 (1)	-	-	1 (1)
Overall HGAEs	13 (11)	6 (1)	35 (17)	2 (1)	31 (12)	87 (42) *

Abbreviations: CSI = craniospinal irradiation; sPBCT = simultaneous radiochemotherapy, * Number of patients in all subgroups without patients received CSI additionally, multiple HGAEs during the same course were recorded, e.g., in total 87 HGAEs occurred in 67 PBT/sPBCT courses. ^#^ Number of patients in supratentorial group without intra-/suprasellar primary tumor site.

**Table 3:** Binary logistic regression analysis for occurrence of HGAEs.

Factor	Significance	Odds Ratio Exp(B)	95% Confidence Interval for Odds Ratio	
			Lower Level	Upper Level
VP shunt	0.004	3.628	1.512	8.706
HD-chemotherapy prior PBT	0.038	5.219	1.092	24.938
Simultaneous CTx to PBT	<0.001	5.631	2.535	12.510
ATRT Histology	0.002	5.808	1.884	17.910
CSI	0.018	2.960	1.201	7.295

VP = ventriculoperitoneal, HD = high dose, PBT = proton beam therapy, ATRT = Atypical teratoid rhabdoid tumor, CSI = craniospinal irradiation.

**Table 4:** Temporary interruptions of proton beam therapy.

Adverse Event Causing PBT Interruptions	Interruptions of PBT (*n*)	Duration of Interruptions (Cumulative Days/ Median Days per Patient)	Major Delays (≥3 Days Interruption of PBT) [*n* (Days)]
Shunt dysfunction	6	32/5	3 (19; 7; 3 days)
Infections	11	28/1	2 (14; 4 days)
Neurotoxicity	4	4/1	-
Non-compliance	2	2/1	-
Anesthesia problems	2	2/1	-
Electrolyte imbalance	1	1/1	-

Abbreviations: PBT = proton beam therapy.

**Table 5:** Binary logistic regression analysis for occurrence of PBT interruptions.

Factor	Significance	Odds Ratio Exp(B)	95% Confidence Interval for Odds Ratio	
			Lower Level	Upper Level
Occurrence of infection	0.049	3.002	1.005	8.971
Occurrence of HGAE	0.005	5.195	1.659	16.267
sPBCT with intensive iv CTx courses	0.184	0.229	0.026	2.016
VP shunt	0.133	2.278	0.778	6.667

CTx = chemotherapy, HGAE = high grade adverse event, iv = intravenous, PBT = proton beam therapy, sPBCT = simultaneous proton beam therapy and chemotherapy, VP = ventriculoperitoneal.

## Data Availability

The anonymized data presented in this study as well as all the software code are available on request from the corresponding authors.
